# Effectiveness of Community-Based Breast Cancer Screening Using Full-Field Digital Mammography With Digital Breast Tomosynthesis in an Urban Population in Central India: An Observational Study

**DOI:** 10.7759/cureus.108117

**Published:** 2026-05-01

**Authors:** Shilpa Pande, Sanjay Dakhore, Deepshikha Arora, Shashikant Juvekar

**Affiliations:** 1 Department of Radiodiagnosis and Interventional Radiology, All India Institute of Medical Sciences, Nagpur, Nagpur, IND; 2 Department of Surgery, Government Medical College, Nagpur, Nagpur, IND; 3 Department of Radiodiagnosis, National Cancer Institute, Nagpur, Nagpur, IND

**Keywords:** bi-rads, breast cancer screening, breast density, breast imaging-reporting and data system, community screening, digital breast tomosynthesis, india, mammography, synthesized 2d imaging

## Abstract

Background

Breast cancer is the most common malignancy among women worldwide and remains a leading cause of cancer-related mortality. In India, delayed presentation and limited access to organised screening programmes contribute to poorer outcomes. Community-based screening models, combined with advanced imaging techniques, may improve early detection. This study evaluates the effectiveness of screening mammography incorporating digital breast tomosynthesis (DBT) with synthesised two-dimensional imaging in a real-world urban population in Central India.

Methods

This community-based cross-sectional screening study involved retrospective analysis of prospectively collected data from 1275 women recruited through outreach programmes in Nagpur, India, between March 2019 and February 2020. All participants underwent full-field digital mammography (FFDM) with DBT. Imaging included two-dimensional craniocaudal views and three-dimensional mediolateral oblique tomosynthesis views, with reconstructed synthesised two-dimensional images for each breast. Breast density, BI-RADS (Breast Imaging Reporting and Data System) categories, and screening outcomes were recorded. Adjunct ultrasonography was performed in selected cases. Screening performance indicators, including cancer detection rate, recall rate, and positive predictive value for biopsy (PPV3), were calculated.

Results

A total of 1275 women were screened, with a mean age of 51 ± 8.09 years, and 95.8% were asymptomatic. Breast density was predominantly ACR Category B (57.4%), followed by Category C (30.2%). The majority of cases were categorised as BI-RADS 1 (75.5%) and BI-RADS 2 (15.6%), while 16 cases (1.3%) were classified as BI-RADS 4 lesions. A statistically significant association was observed between breast density and BI-RADS categories (p = 0.018), with higher BI-RADS categories more frequent in women with heterogeneously dense breasts. No significant association was found between age group and BI-RADS categories (p = 0.21). Histopathological correlation was available in four cases, with two confirmed malignancies. The cancer detection rate was 1.6 per 1000 women screened, the recall rate was 8.5%, and the PPV3 for biopsy was 50%.

Conclusion

Community-based breast cancer screening using FFDM combined with DBT and synthesised imaging is feasible and clinically effective in an Indian setting. DBT enhances the detection of small and occult lesions, particularly in women with dense breasts. Strengthening follow-up systems and expanding structured, imaging-based screening programmes would significantly improve early detection and outcomes in resource-limited settings.

## Introduction

Breast cancer is the most frequently diagnosed malignancy among women worldwide and remains a major contributor to cancer-related morbidity and mortality. According to global cancer statistics, it accounts for over 2.3 million new cases annually and continues to be a leading cause of cancer-related deaths [1,2]. The burden of breast cancer is increasing across both developed and developing regions, with a particularly rapid rise observed in low- and middle-income countries [3,4]. Early detection is a critical determinant of improved outcomes, as cancers diagnosed at an early stage are associated with better prognosis, less aggressive treatment, and reduced mortality [4,5].

In India, breast cancer has emerged as the leading cancer among women, with a steadily increasing incidence over recent decades [6]. Several sociocultural and systemic factors contribute to delayed diagnosis, including lack of awareness, fear of cancer, social stigma, and limited access to healthcare services [7]. As a result, a substantial proportion of patients present at advanced stages, leading to poorer survival outcomes compared to Western populations [8]. Furthermore, the absence of organised population-based screening programmes and low uptake of screening mammography continue to exacerbate this burden.

In resource-limited settings, breast cancer screening strategies are often based on breast self-examination and clinical breast examination. While these approaches are cost-effective and improve awareness, they have limited sensitivity for detecting early-stage disease [9,10]. In contrast, screening mammography is widely recognised as the most effective method for early detection of breast cancer. Evidence from large randomised trials, including the Swedish Two-County Trial, has demonstrated a significant reduction in breast cancer mortality with mammographic screening [11,12].

Breast density plays an important role in the effectiveness of screening, as dense fibroglandular tissue not only increases the risk of malignancy but also reduces the sensitivity of mammography due to masking of lesions [13,14]. Advances in imaging technology have led to the development of digital breast tomosynthesis (DBT), a three-dimensional imaging modality that improves lesion detection by reducing tissue overlap. In addition, synthesised two-dimensional images reconstructed from tomosynthesis datasets allow evaluation of conventional projections without additional radiation exposure, thereby improving workflow efficiency while maintaining diagnostic accuracy. Several studies have demonstrated that DBT, particularly when combined with synthesised imaging, improves cancer detection rates and reduces recall rates compared to conventional mammography [15-17].

Despite these advances, there remains a paucity of real-world data on the effectiveness of community-based mammographic screening programmes in India, particularly those incorporating DBT and synthesised imaging. Most available studies are based on hospital populations and do not accurately represent true screening cohorts. Furthermore, challenges related to accessibility, awareness, and follow-up continue to limit the effectiveness of screening initiatives in community settings. Generating such evidence is essential to inform future screening policies and optimise their effectiveness.

In this context, the present study was conducted to evaluate the effectiveness of community-based breast cancer screening using full-field digital mammography (FFDM) combined with DBT in an urban population in Central India, based on ACR BI-RADS guidelines [18]. This study aims to evaluate the distribution of BI-RADS categories, characterise breast density patterns, and analyse key screening performance metrics, including cancer detection rate and recall rate.

## Materials and methods

This was a community-based cross-sectional screening study involving retrospective analysis of prospectively collected data conducted under the Ayushmati project in Nagpur, India, between March 2019 and February 2020. Screening camps were organized weekly, facilitating participation from the surrounding urban population.

Participants were recruited through structured outreach strategies, including awareness campaigns, advertisements, and mobilisation by community health workers under the National Urban Health Mission. Written informed consent was obtained from all participants. Women aged 40 years and above undergoing routine screening, as well as younger women with a first-degree family history of breast cancer, were included. Women with a prior history of breast cancer, those undergoing treatment for breast cancer, or those with incomplete imaging data were excluded. A total of 1275 women were included in the final analysis.

All participants underwent a standardised screening protocol that included clinical breast examination followed by imaging. Screening mammography was performed using FFDM integrated with DBT on a Senographe Pristina (Hologic Inc., Marlborough, MA). Bilateral imaging included two-dimensional craniocaudal views obtained using FFDM and three-dimensional mediolateral oblique tomosynthesis views. Synthesised two-dimensional mediolateral oblique images were reconstructed from the tomosynthesis dataset for each breast, eliminating the need for additional conventional exposures.

This imaging protocol was designed to optimise diagnostic performance while maintaining radiation safety. The use of synthesised two-dimensional images reduces cumulative radiation dose without compromising diagnostic accuracy. The overall radiation exposure remained within internationally accepted safety limits, consistent with established screening guidelines [15,17].

All images were interpreted by experienced radiologists in accordance with the American College of Radiology Breast Imaging Reporting and Data System (BI-RADS), 5th edition, as described in Table 1 [18]. Breast density was categorised into ACR categories A through D. Adjunct USG (ultrasonography) was performed using a GE LOGIQ E9 (GE Healthcare, Chicago, IL) in women with dense breasts and in those with BI-RADS category 3 or higher findings.

**Table 1:** Breast imaging assessment categories and recommended management as per the ACR BI-RADS Adapted BI-RADS assessment categories with corresponding management recommendations and estimated malignancy risk (adapted from BI-RADS lexicon [18]). This table represents a restructured version of the original classification, solely for academic and illustrative purposes. ACR: American College of Radiology; BI-RADS: Breast Imaging Reporting and Data System

Category	Interpretation	Suggested Management	Approximate Malignancy Risk
0	Assessment is incomplete; additional evaluation is needed, especially when prior studies are unavailable	Perform further imaging and/or compare with previous examinations	Not applicable
1	No imaging abnormality identified	Continue with regular screening protocol	~0%
2	Findings are definitively benign	Routine screening advised	~0%
3	Probably benign lesion	Short-term follow-up imaging recommended (usually at 6 months)	>0% to ≤2%
4	Suspicious finding with a possibility of malignancy	Biopsy should be considered for histological confirmation	Subcategories: 4a (2–10%), 4b (10–50%), 4c (50–95%)
5	Imaging strongly suggests malignancy	Tissue diagnosis is strongly recommended	>95%
6	Malignancy already proven on prior biopsy	Proceed with appropriate definitive treatment (e.g., surgery or oncologic management)	Not applicable

Women categorised as BI-RADS 4 or higher were advised of histopathological evaluation. Outcome measures included breast density characterisation, BI-RADS distribution, cancer detection rate, recall rate, and positive predictive value for biopsy (PPV3). All representative images in this study originate from our institutional patient cohort.

Statistical analysis

Statistical analysis was performed using STATA version 14.0 (StataCorp, College Station, TX). Descriptive statistics were used to summarise the data. Continuous variables were expressed as mean ± standard deviation (SD) and median where appropriate, while categorical variables were presented as frequencies and percentages (n, %). Association between categorical variables, including BI-RADS categories with age groups and breast density, was assessed using Fisher’s exact test due to small expected cell counts in several categories. A p-value of <0.05 was considered statistically significant. For tables with single-variable distributions or insufficient sample size (e.g., histopathological correlation subset), only descriptive statistics were reported, and no inferential statistical tests were applied. As Fisher’s exact test was used, no test statistic value was reported.

Ethical approval

Ethical approval for this study was obtained from the Institutional Ethics Committee of the National Cancer Institute, Nagpur. The study involved retrospective analysis of prospectively collected data. Written informed consent was obtained from all participants at the time of enrolment.

## Results

A total of 1275 women were included in the study and constituted the final screening cohort. The majority of women belonged to the 40-60-year age group (mean age of 51 ± 8.09 years), accounting for 1048 of 1275 participants (82.2%). The distribution of BI-RADS categories across different age groups is presented in Table 2. Most participants were asymptomatic (N=1221; 95.8%), reflecting a true screening population. A small subset of women reported non-specific or vague symptoms such as breast pain or menstrual complaints associated with breast discomfort (N=54; 4.2%). Most of them were categorised as BI-RADS 1 or 2 across all age groups. No statistically significant association was observed between age group and BI-RADS categories.

**Table 2:** Distribution of BI-RADS categories across age groups Data presented as n (%). Association assessed using Fisher’s exact test (p = 0.21). BI-RADS: Breast Imaging Reporting and Data System

Age group	BI-RADS 1	BI-RADS 2	BI-RADS 3	BI-RADS 4a	BI-RADS 4b	BI-RADS 4c	Total
<40	8 (80%)	2 (20%)	0	0	0	0	10
40–49	478 (75.7%)	86 (13.6%)	59 (9.3%)	6 (0.9%)	1 (0.2%)	1 (0.2%)	631
50–59	311 (74.6%)	68 (16.3%)	31 (7.4%)	1 (0.2%)	4 (1.0%)	2 (0.5%)	417
60–69	145 (77.1%)	38 (20.2%)	5 (2.7%)	0	0	0	188
≥70	22 (75.9%)	5 (17.2%)	2 (6.9%)	0	0	0	29

**Figure 1:**
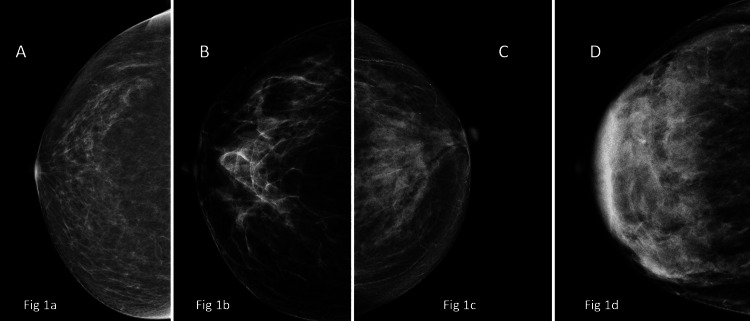
Representative images of various breast densities as per ACR BI-RADS Category A: almost entirely fatty (Fig 1a); Category B: scattered areas of fibroglandular tissue (Fig 1b); Category C: Heterogeneously dense breast (Fig 1c); Category D: Extremely dense breast (Fig 1d). ACR: American College of Radiology; BI-RADS: Breast Imaging Reporting and Data System

Breast density was predominantly ACR Category B (N=732, 57.4%), followed by Category C (N=385, 30.2%), with fewer women in Categories A (N=148, 11.6%) and D (N=10, 0.8%) (Table 3). Representative mammographic images illustrating ACR breast density categories A through D are shown in Figure 1.

**Table 3:** Breast density distribution (ACR categories) ACR: American College of Radiology

Density Category	Number (%)
A	148 (11.6%)
B	732 (57.4%)
C	385 (30.2%)
D	10 (0.8%)
Total	1275 (100%)

**Figure 2:**
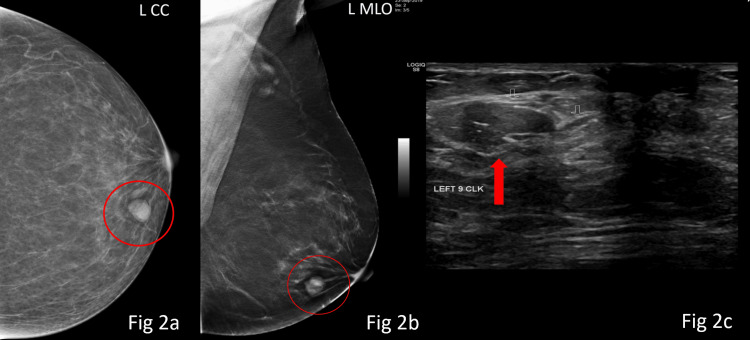
A well-defined, round, high-density lesion is seen in the lower inner quadrant of the left breast (red circle in Figs 2a-2b). On ultrasonography (Fig 2c), it corresponds to a well-circumscribed, hypoechoic solid intraductal lesion, categorised as BI-RADS 4a. Histopathological examination confirmed the diagnosis of a benign intraductal papilloma. BI-RADS: Breast Imaging Reporting and Data System

**Figure 3:**
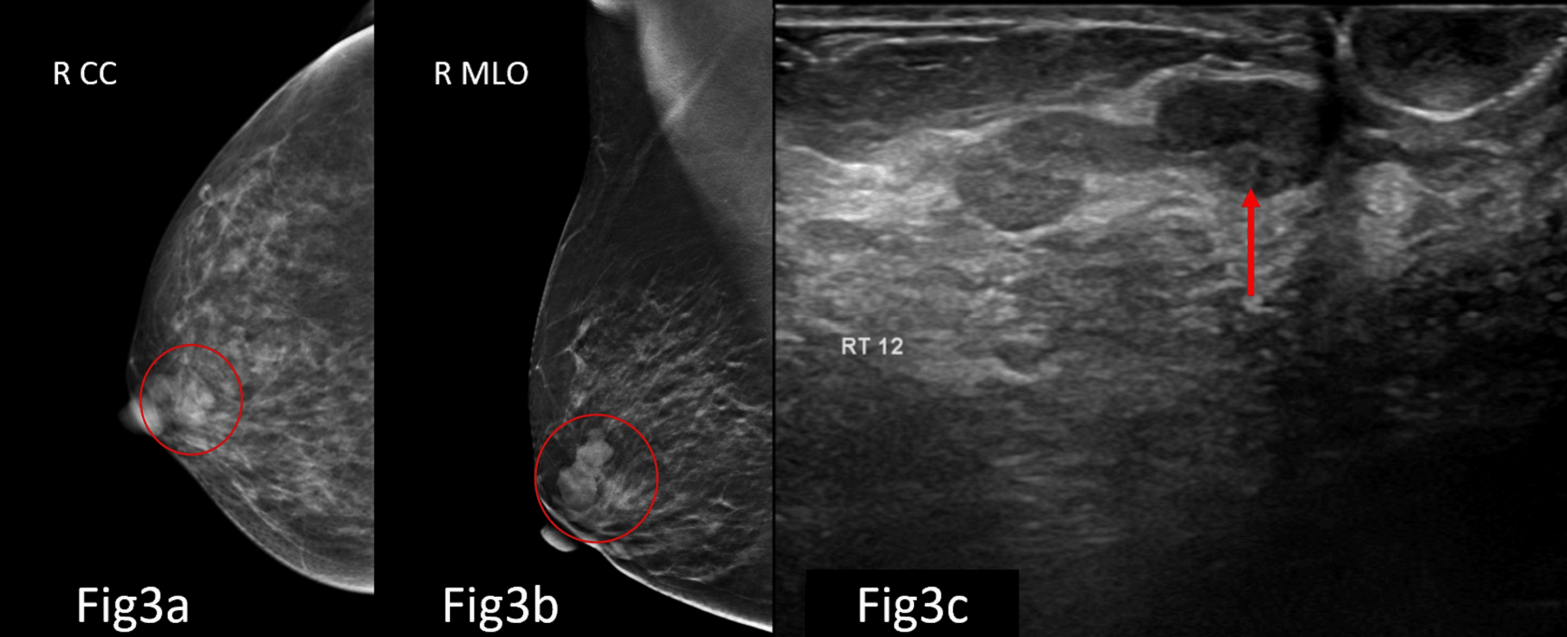
Mammography CC and MLO views (Fig 3a-3b) showed a well-circumscribed, high-density mass in the periareolar region of the right breast. No suspicious calcifications or architectural distortion were seen. On USG correlation (Fig 3c), a well-circumscribed, hypoechoic, non-infiltrating, wider than taller, solid intraductal mass lesion was noted. It was categorised as BI-RADS 4a, indicating low suspicion for malignancy, and histopathological correlation revealed benign breast disease. BI-RADS: Breast Imaging Reporting and Data System

**Figure 4:**
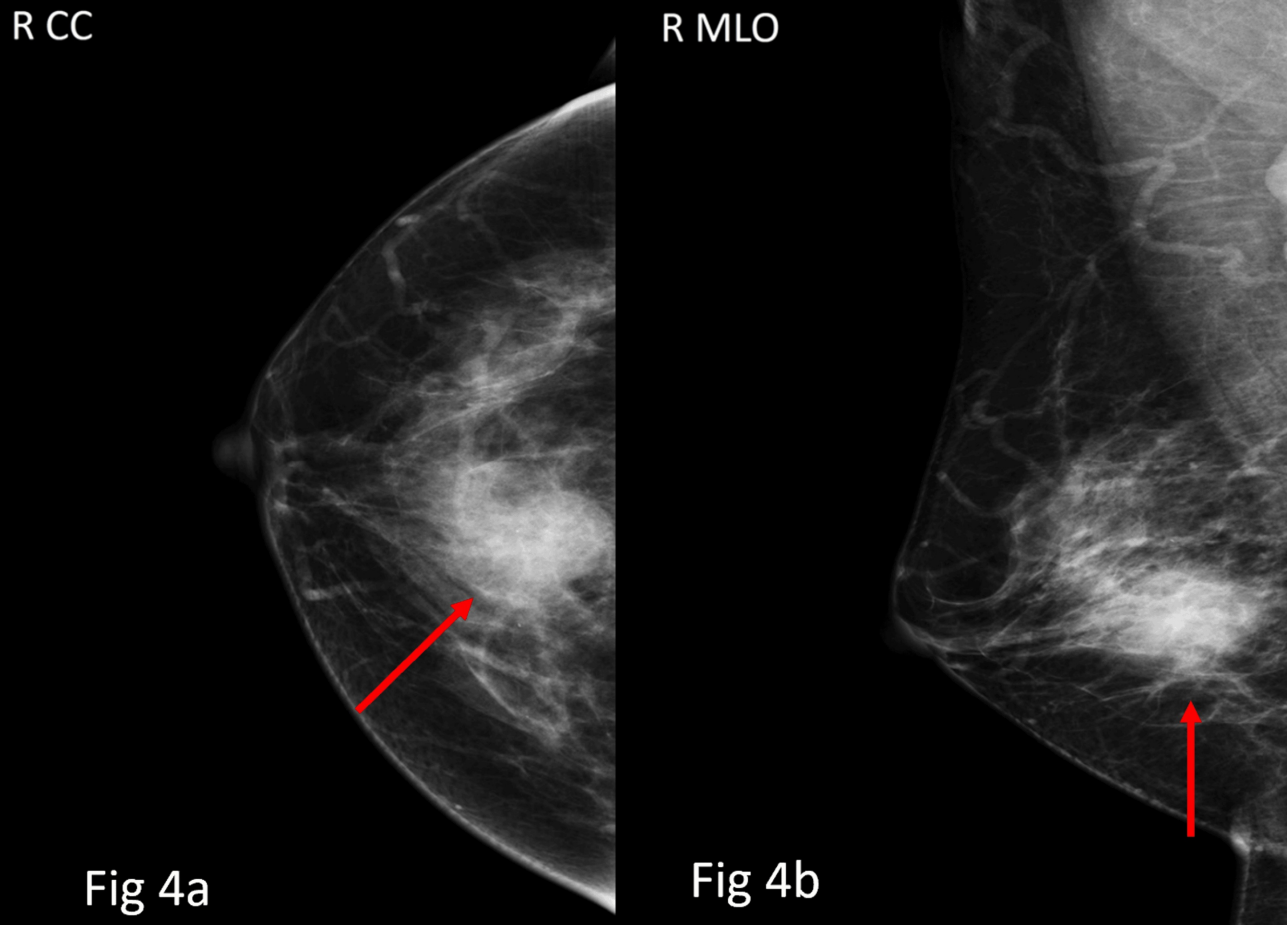
On mammography, MLO and CC view (Figs 4a-4b), there is an irregular high-density lesion with partly obscured margins in the lower inner quadrant of the right breast. The lesion demonstrated associated architectural distortion without definite calcifications. It was categorised as BI-RADS 4b, indicating moderate suspicion for malignancy. BI-RADS: Breast Imaging Reporting and Data System

**Figure 5:**
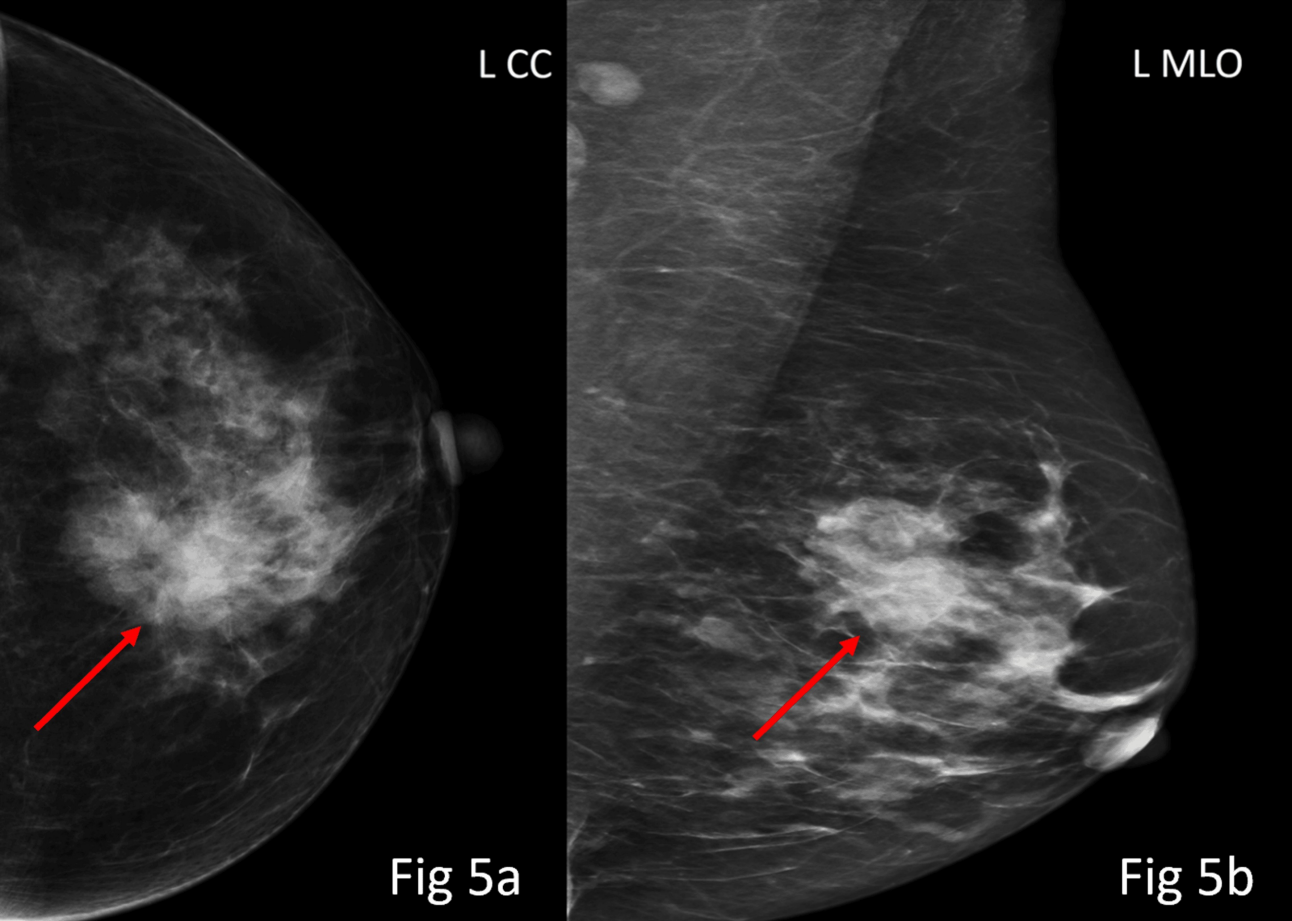
On mammography CC and MLO views (Figs 5a-5b), there is a high-density mass in the inner central region of the left breast (red arrows), demonstrating irregular shape with partly microlobulated and partly indistinct margins. No suspicious calcification was seen. The lesion was categorised as BI-RADS 4c, indicating high suspicion for malignancy, and histopathological correlation revealed invasive ductal carcinoma Grade III. BI-RADS: Breast Imaging Reporting and Data System

Overall, the majority of cases were categorised as BI-RADS 1 category, 963 (75.5%), and BI-RADS 2 category, 199 (15.6%), while BI-RADS 3 accounted for 97 (7.6%) cases. A total of 16 (1.3%) patients had BI-RADS 4 lesions (4a: 7 (0.5%); 4b: 5 (0.4%); 4c: 4 (0.3%)), with no BI-RADS 5 lesions identified, 0 (0%) (Table 4). Representative examples of BI-RADS 4a (Figures 2-3), 4b (Figure 4), and 4c (Figure 5) lesions from our cohort are shown below.

**Table 4:** Distribution of BI-RADS categories BI-RADS: Breast Imaging Reporting and Data System

BI-RADS Category	Number (%)
1	963 (75.5%)
2	199 (15.6%)
3	97 (7.6%)
4a	7 (0.5%)
4b	5 (0.4%)
4c	4 (0.3%)
5	0 (0%)
Total	1275 (100%)

The distribution between BI-RADS categories and breast density is illustrated in Table 5. A statistically significant association was observed between breast density and BI-RADS categories (Fisher’s exact test, p = 0.018), with higher BI-RADS categories more frequently seen in women with heterogeneously dense breasts (ACR Category C).

**Table 5:** Distribution of BI-RADS categories across breast density groups Data presented as n (% row-wise). Association assessed using Fisher’s exact test (p = 0.018), indicating a statistically significant association. BI-RADS: Breast Imaging Reporting and Data System

BI-RADS	A	B	C	D	Total
1	114 (11.8%)	597 (62.0%)	246 (25.5%)	6 (0.6%)	963
2	31 (15.6%)	92 (46.2%)	74 (37.2%)	2 (1.0%)	199
3	3 (3.1%)	36 (37.1%)	56 (57.7%)	2 (2.1%)	97
4a	0	2 (28.6%)	5 (71.4%)	0	7
4b	0	3 (60.0%)	2 (40.0%)	0	5
4c	0	2 (50.0%)	2 (50.0%)	0	4

Histopathological correlation was available in four patients, of whom two were malignant and two were benign. Given the exploratory nature of this subgroup, formal inferential statistical testing was not undertaken. Data was analysed descriptively with emphasis on observed trends (Table 6).

**Table 6:** Distribution of histopathological findings in BI-RADS 4 lesions Histopathological evaluation was available only in limited subset of cases. Due to the small sample size (n=4), no inferential statistical test was applied. Therefore, the findings are descriptive in nature. BI-RADS: Breast Imaging Reporting and Data System

BI-RADS	Benign	Malignant
4a (n=7)	2	0
4b (n=5)	0	0
4c (n=4)	0	2

**Figure 6:**
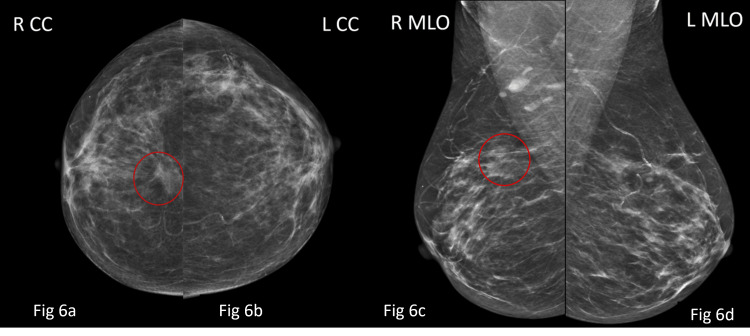
Mammography images of both right and left breast in CC (Fig 6a, 6b) and MLO views (Fig 6c, 6d) show an irregular high density mass (red circles) with indistinct margins and associated architectural distortion and no suspicious intra-lesional calcifications in central region of retro-mammary zone of right breast. However, no obvious lesion was seen in left breast. Lesion in right breast was categorised as BI-RADS 4c, indicating highly suspicious for malignancy. BI-RADS: Breast Imaging Reporting and Data System

**Figure 7:**
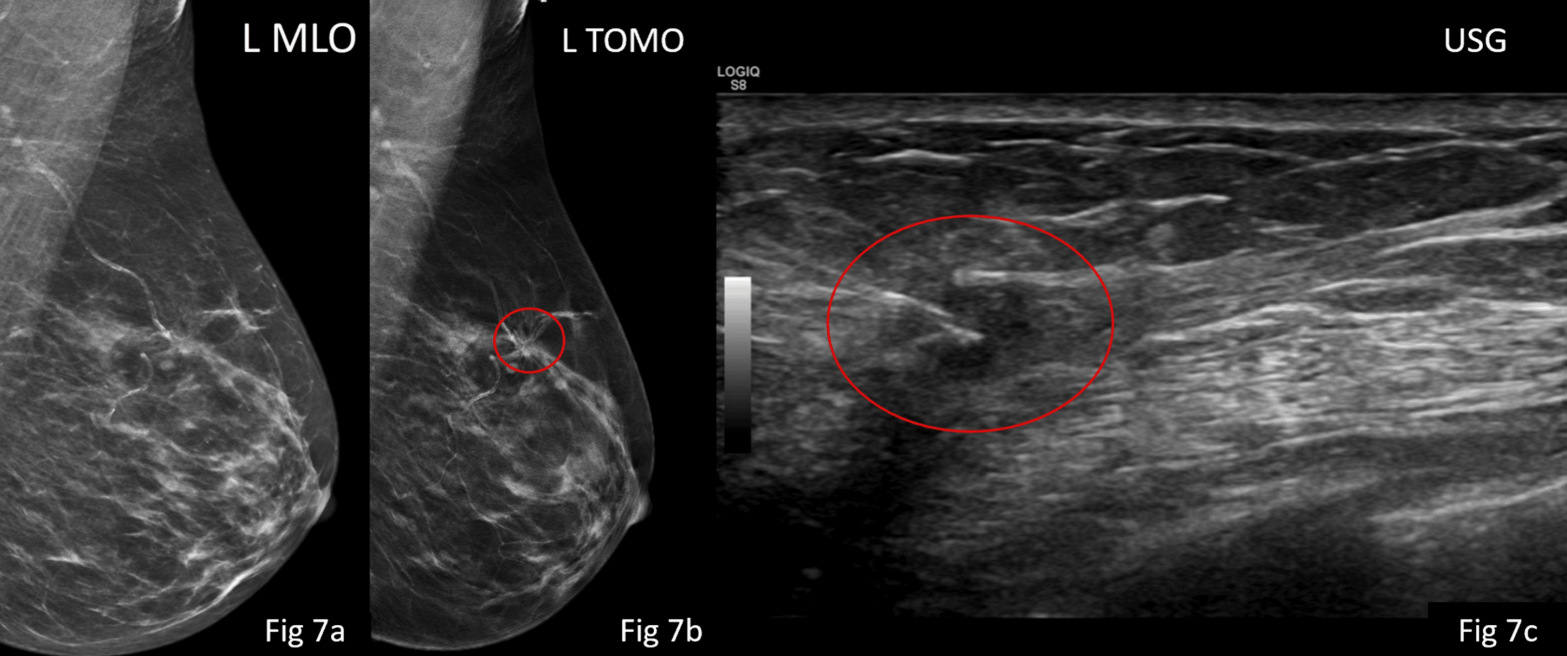
Mammography 2D-synthesised MLO (Fig 7a) view of the left breast shows no obvious mass; however, on tomography (Fig 7b), a high density focal architectural distortion was seen in upper aspect of left breast. On USG, an irregular hypoechoic lesion of size 8mm (maximum dimension) was seen and was characterised as a 4c lesion. It was thereafter biopsied (needle track demonstrated in Fig 7c) and turned out to be duct carcinoma.

Among the malignant cases, one patient had bilateral breast carcinoma with both lesions categorised as BI-RADS 4c. In this patient, an approximately 8 mm lesion in the contralateral breast was detected exclusively on DBT and was not visualised on synthesised two-dimensional imaging, and was subsequently confirmed as malignant on histopathology. Mammographic and tomosynthesis findings of this case are illustrated in Figures 6-7.

The cancer detection rate was 1.6 per 1000 women screened, the recall rate was 8.5%, and the PPV3 for biopsy was 50%. A proportion of patients with BI-RADS 4 lesions were lost to follow-up, which may have led to underestimation of the true cancer detection rate.

## Discussion

This study provides real-world evidence supporting the feasibility and effectiveness of community-based breast cancer screening using DBT in an urban population in Central India. A key strength of this study is the inclusion of a predominantly asymptomatic cohort, which closely reflects a true screening population and minimises selection bias commonly associated with hospital-based studies. In established screening programmes, asymptomatic populations typically demonstrate a predominance of BI-RADS 1 and 2 findings, a pattern that was also observed in our cohort. This is consistent with findings from large population-based screening trials, where the majority of screened women demonstrate benign or negative results [11,19].

The age distribution in this study highlights the relatively earlier occurrence of breast abnormalities in Indian women compared to Western populations, with the majority of participants falling within the 40-60-year age group [8]. Although higher BI-RADS categories were more frequently observed in this group, the association between age and BI-RADS categories was not statistically significant (p = 0.21). This suggests that, within the screened cohort, age alone may not be a strong independent determinant of imaging categorisation, although it remains an important epidemiological factor.

Breast density emerged as a critical factor influencing screening outcomes in this study. A statistically significant association was observed between breast density and BI-RADS categories (p = 0.018), with higher BI-RADS categories more frequently seen in women with heterogeneously dense breast parenchyma (ACR Category C). Increasing fibroglandular density can reduce lesion conspicuity due to overlapping tissue, thereby masking underlying abnormalities and posing a challenge in image interpretation. These findings are consistent with prior studies that have established breast density as both a risk factor for malignancy and a limiting factor for mammographic sensitivity [13,14].

A particularly important observation in this study was the detection of a small lesion exclusively on DBT, which was not visualised on either synthesised two-dimensional imaging or conventional mammography. This finding highlights the capability of DBT to overcome the limitations of tissue superimposition by providing quasi-three-dimensional imaging, thereby enhancing lesion conspicuity, particularly in dense breast parenchyma. Previous studies have demonstrated that DBT improves cancer detection rates while maintaining acceptable recall rates, and our findings provide real-world support for its incremental diagnostic value in a community-based screening setting [15,17].

The cancer detection rate observed in this study (1.6 per 1000 women screened) is lower than that reported in organised Western screening programmes, which typically range from 4 to 8 per 1000 women [11]. However, a population-based screening study from Punjab using artificial intelligence-assisted thermal imaging reported a comparable detection rate of approximately 1.8 per 1000 women screened [20]. These differences may reflect variations in screening modality, population characteristics, and follow-up patterns.

The recall rate of 8.5% observed in this study falls within internationally accepted benchmarks (5-10%) and is comparable to those reported in large screening trials [11]. The use of DBT may have contributed to maintaining an acceptable recall rate while improving lesion visualisation, particularly in women with dense breasts. This is consistent with emerging evidence supporting the role of tomosynthesis in improving screening performance [21,22]. Adjunct ultrasonography may also be useful in selected women with dense breasts or indeterminate findings on mammography [23]; however, its incremental value was not specifically assessed in the present study.

An important observation in this study was that clinical symptoms were not consistently reflective of imaging findings. Many women presenting with breast-related complaints had normal or benign imaging results, while malignancies were detected in asymptomatic individuals. These findings highlight the limitations of symptom-based evaluation and reinforce the importance of structured, imaging-based screening approaches.

From a broader public health perspective, community-based screening initiatives play a crucial role in improving accessibility, increasing awareness, and facilitating early detection, particularly in resource-limited settings. Such programmes have the potential to shift the stage at diagnosis and ultimately improve survival outcomes at a population level. To our knowledge, this study represents one of the few community-based evaluations in India integrating DBT into a structured screening framework, thereby providing valuable real-world evidence to inform future screening strategies and public health policy.

Limitations

This study has certain limitations. Histopathological correlation was available in only a small subset of patients, which may affect the accuracy of calculated screening performance metrics. A proportion of patients with BI-RADS 4 lesions were lost to follow-up, potentially leading to underestimation of the true malignancy burden. The absence of longitudinal follow-up limits the assessment of interval cancers and long-term outcomes such as mortality reduction. Additionally, a direct comparison between DBT and conventional two-dimensional mammography was not performed. As a single-center study conducted in an urban population, the findings may have limited generalizability to other settings.

## Conclusions

This study demonstrates that community-based breast cancer screening using FFDM combined with DBT and synthesised two-dimensional imaging is both feasible and clinically effective in an urban population in Central India. The findings highlight that a predominantly asymptomatic cohort can be successfully screened, with imaging outcomes comparable to established screening benchmarks.

The study reflects the critical influence of breast density on diagnostic performance and reinforces the incremental value of DBT in detecting small and otherwise occult malignancies, as evidenced by the identification of a DBT-only lesion. Despite a relatively low cancer detection rate, the acceptable recall rate and positive predictive value indicate balanced and effective screening performance. Importantly, the lack of correlation between symptoms and malignancy emphasises the need to move beyond symptom-based evaluation toward structured, population-based screening strategies. From a public health perspective, community-based screening programmes have the potential to improve accessibility, increase awareness, and facilitate earlier diagnosis.

Overall, these findings support the expansion of organised, imaging-based breast cancer screening programmes in India, with emphasis on early initiation, imaging strategies considering the factor of breast density, and strengthened follow-up mechanisms to optimise patient outcomes.
